# Proteomic characterization of *Mycobacterium tuberculosis* subjected to carbon starvation

**DOI:** 10.1128/msystems.01530-24

**Published:** 2025-04-15

**Authors:** Kaylyn L. Devlin, Damon T. Leach, Kelly G. Stratton, Gyanu Lamichhane, Vivian S. Lin, Kimberly E. Beatty

**Affiliations:** 1 Department of Chemical Physiology and Biochemistry, Oregon Health and Science University, Portland, Oregon, USA; 2 Biological Sciences Division, Pacific Northwest National Laboratory 6865, Richland, Washington, USA; 3 Division of Infectious Diseases, Department of Medicine, Johns Hopkins University, Baltimore, Maryland, USA; University of Southampton, Southampton, United Kingdom

**Keywords:** *Mycobacterium tuberculosis*, dormancy, infectious disease, proteomics, antibiotic resistance, metabolism

## Abstract

*Mycobacterium tuberculosis* (*Mtb*) is the causative agent of tuberculosis (TB), the leading cause of infectious disease-related deaths worldwide. TB infections present on a spectrum from active to latent disease. In the human host, *Mtb* faces hostile environments, such as nutrient deprivation, hypoxia, and low pH. Under these conditions, *Mtb* can enter a dormant, but viable, state characterized by a lack of cell replication and increased resistance to antibiotics. Dormant *Mtb* poses a major challenge to curing infections and eradicating TB globally. We subjected *Mtb* mc^2^6020 (Δ*lysA* and Δ*panCD*), a double auxotrophic strain, to carbon starvation (CS), a culture condition that induces growth stasis and mimics environmental conditions associated with dormancy *in vivo*. We provide a detailed analysis of the proteome in CS compared to replicating samples. We observed extensive proteomic reprogramming, with 36% of identified proteins significantly altered in CS. Many enzymes involved in oxidative phosphorylation and lipid metabolism were retained or more abundant in CS. The cell wall biosynthetic machinery was present in CS, although numerous changes in the abundance of peptidoglycan, arabinogalactan, and mycolic acid biosynthetic enzymes likely result in pronounced remodeling of the cell wall. Many clinically approved anti-TB drugs target cell wall biosynthesis, and we found that these enzymes were largely retained in CS. Lastly, we compared our results to those of other dormancy models and propose that CS produces a physiologically distinct state of stasis compared to hypoxia in *Mtb*.

**IMPORTANCE:**

Tuberculosis is a devastating human disease that kills over 1.2 million people a year. This disease is caused by the bacterial pathogen *Mycobacterium tuberculosis* (*Mtb*). *Mtb* excels at surviving in the human host by entering a non-replicating, dormant state. The current work investigated the proteomic changes that *Mtb* undergoes in response to carbon starvation, a culture condition that models dormancy. The authors found broad effects of carbon starvation on the proteome, with the relative abundance of 37% of proteins significantly altered. Protein changes related to cell wall biosynthesis, metabolism, and drug susceptibility are discussed. Proteins associated with a carbon starvation phenotype are identified, and results are compared to other dormancy models, including hypoxia.

## INTRODUCTION

Tuberculosis (TB) is the deadliest infectious disease in human history. *Mycobacterium tuberculosis* (*Mtb*), the underlying bacterial pathogen, causes over 10 million infections and over 1.2 million deaths per year. After more than a decade of slow decline in TB incidence, global case numbers started to increase in 2020 with the onset of the COVID-19 pandemic (1). Unfortunately, the international effort to end the TB pandemic by 2030 remains uncertain and challenging (2).

TB can present as an active, symptomatic disease or an asymptomatic latent TB infection (LTBI). An estimated 25% of the global population has an LTBI, with the potential to progress to an active infection at a later time (1). A hallmark of LTBI is *Mtb*’s unusual ability to survive within the host indefinitely in a non-replicating persistent state known as dormancy. Additionally, patients with active TB harbor physiologically distinct subpopulations of *Mtb* across a spectrum from dormant to active (3), highlighting that infections are complex and heterogeneous. Dormant bacteria are challenging to kill in the host because they are phenotypically drug resistant (4).

There are a variety of *in vitro* models that mimic host environments associated with *Mtb* dormancy (4–18). Bacteria are subjected to hypoxia, nutrient deprivation, low pH, reactive nitrogen species, alternative carbon sources, or a combination of these stressors. A feature of most models is that *Mtb* enters stasis, where growth is negligible over time. Upon entering stasis, *Mtb* undergoes widespread transcriptional and proteomic changes leading to the reorganization of many cellular processes (18, 19). *Mtb* slows metabolism and becomes phenotypically resistant to most drugs (20). The most common dormancy model, the Wayne model, uses gradually induced hypoxia to shift *Mtb* into two distinct stages of non-replicating persistence (11). This model is well described through multiple transcriptomic and proteomic studies (7–10). The nutrient starvation model was first described in 1933 by Loebel (6) and later developed and characterized by Betts et al. (13). The Betts model cultures *Mtb* in carbon- and lipid-free buffer for 6 weeks. A closely related carbon starvation (CS) model was described by Grant and coworkers (5). Multi-stress models have also been described (14, 15). Occasionally, the stationary phase is used as a surrogate for dormancy (16, 17). The choice of the model is usually based on the aspect of TB disease that the researcher intends to study.

No single *in vitro* model is likely to recapitulate all features of dormant *Mtb* found in a host environment, especially since microenvironments vary within a single host (3). However, these models allow us to study the effects of validated dormancy-associated conditions on *Mtb* biology and drug response (21). There have been a variety of proteomic and transcriptomic studies on *Mtb* dormancy models, as recently reviewed (18). However, nearly all have focused on dormancy induced by hypoxia. Nutrient deprivation is likely encountered by *Mtb in vivo*, particularly within the granuloma. The CS model captures many observed features of *Mtb* isolated from patients, including loss of acid-fast staining, drug tolerance, and low respiration (22). CS is also easier to implement in the lab and more reproducible for drug screening compared to hypoxia (5).

Before now, there was limited information on proteomic changes associated with nutrient or carbon starvation (13, 23). The most recent study was published over a decade ago and focused solely on secreted proteins (i.e., culture filtrates) during nutrient starvation (23). Therefore, we sought to characterize proteomic changes in *Mtb* under CS conditions. We identified specific proteins and their abundance changes between replicating and CS conditions. Our results provide a comprehensive overview of protein-level adaptations used by *Mtb* to survive carbon starvation. We focus our analysis on changes in metabolism, cell wall biosynthesis, and drug targets.

## MATERIALS AND METHODS

### Experimental design and statistical rationale

A total of 12 samples were analyzed in this study, including six biological replicates in two distinctly cultured groups: replicating (Rep; *n* = 6) and CS (*n* = 6). The number of replicates was determined for sufficient power in quantitation and statistical analyses (24). Samples were analyzed by liquid chromatography tandem mass spectrometry (LC-MS/MS) in randomized order.

### Mycobacterial culture conditions

*Mtb* mc^2^6020 (Δ*lysA* and Δ*panCD*) (25), a double auxotrophic mutant derived from the laboratory strain *Mtb* H37Rv, was obtained from W. Jacobs’s laboratory (Albert Einstein College of Medicine and HHMI). It was handled as a BSL-2 pathogen under Oregon Health & Science University-approved biosafety protocols. All bacterial manipulation was done within a biosafety cabinet to minimize the potential for exposure to the pathogen.

Bacteria were thawed from frozen stocks stored at −80°C in 30% glycerol. *Mtb* was cultured in 7H9/OADC-KPC medium (7H9 broth [BD Difco], 0.5% glycerol [Fisher, molecular biology grade], 0.05% Tween 80 [Sigma], 10% OADC [BD Difco], 80 µg/mL lysine [K; Sigma], 24 µg/mL pantothenate [P, Sigma], and 0.2% casamino acids [C, Gibco]). Cultures were grown at 37°C with 100 rpm in aerated polycarbonate shake flasks with a 0.2 µm filter cap (Weaton #WPFPC0500S).

Culture conditions to induce dormancy via CS were adapted from previously reported methods (5, 26). *Mtb* was grown in 7H9/OADC-KPC to an OD_600_ of 0.8–1.2. Cells were washed twice with phosphate buffered saline (PBS) and diluted to an OD_600_ of 0.2 in carbon starvation medium (7H9/Tx-KP; 7H9 broth, 0.05% tyloxapol [Sigma], 80 µg/mL lysine, and 24 µg/mL pantothenate). Cultures (*n* = 6, 300 mL) were grown standing at 37°C in 1 L plug-sealed bottles (Corning #430195) for 5 weeks (Table S1).

Matched replicating cultures were simultaneously prepared from the same washed cell stock. Cells were diluted to an OD_600_ of 0.2 in 7H9/OADC-KPC medium. Cultures (*n* = 6, 200 mL) were grown shaking (100 rpm, 37°C) in aerated 500 mL shake flasks until an OD_600_ of ~1.0 was reached. Cells were harvested through centrifugation (5 min, 4,000 × *g*, 4°C), washed twice with PBS, and stored at −30°C in PBS until lysis.

### Lysate preparation

Cells were lysed following a protocol adapted from previously described methods (27). Frozen cell pellets were thawed on ice. Cells were lysed in PBS by mechanical disruption with a MiniLys Beadbeater (Bertin Technologies) at 5,000 rpm (3 × 45 s, cooling between on ice for 180 s) using 0.1 mm zirconia/silica beads (BioSpec Products). Beads and cell debris were pelleted by centrifugation (18,000 × *g*, 5 min, 4°C). After the first clarified supernatant was collected, 1 mL of 1% n-dodecyl-D-β-maltoside (Chem-Impex #21950, CAS 69227-93-6) in PBS (PBS-DM) was added to the remaining beads and cell debris. The slurry was resuspended and incubated for 30 min on ice. The second supernatant was collected after centrifugation and added to the clarified supernatant to give lysate in buffer with a final concentration of 0.5% n-dodecyl-D-β-maltoside. Total lysates were centrifuged one final time to remove residual beads (18,000 × *g*, 5 min, 4°C). Lysates were filtered twice through 0.2 µm polyvinylidene fluoride (PVDF) membrane filters (13 mm, Pall) to sterilize. The second filtration was done in a sterilized biosafety cabinet. A bicinchoninic acid (BCA) assay (Pierce) was used to quantify the total protein concentration of all lysates.

### Mass spectrometry sample preparation

Fresh lysate proteins (150 µg) were digested with proteomics-grade trypsin (Promega) overnight at room temperature (RT) with end-over-end rotation. Solid phase extraction (SPE) C18 columns (50 mg bed wt., Millipore-Sigma Supelco Discovery DSC-18) were conditioned with methanol (3 mL, under vacuum, Fisher) and rinsed with acidified water (2 mL, 0.1% trifluoroacetic acid [TFA], Fisher). The peptides from the digestion were applied to the SPE columns and slowly allowed to pass through the column (≤1 mL/min). Samples were washed with 4 mL of 95:5 H_2_O:acetonitrile (ACN; Fisher) with 0.1% TFA. Columns were allowed to go to dryness, and column tips were wiped to remove any residue. Peptides were slowly eluted from columns with 20:80 H_2_O:ACN with 0.1% TFA (1 mL), under vacuum. The samples were dried in a SpeedVac concentrator and resuspended in high performance liquid chromatography (HPLC)-grade water (50 µL). Peptide concentrations were quantified via BCA assay. Samples were normalized to 50 µL of 0.1 µg/µL of total peptide, and 30 µL of each sample was transferred to vials (MicroSolv 2 mL vials with AQ Brand deactivated glass low volume inserts) for MS analysis.

### Liquid chromatography tandem mass spectrometry

LC-MS/MS analysis of global proteomics samples was conducted using a Waters nanoAcquity ultra-performance liquid chromatography system connected to a Q Exactive Plus Orbitrap mass spectrometer (Thermo Scientific). Samples were loaded into a precolumn (150 µm i.d., 4 cm length) packed in-lab with Jupiter C18 packing material (300 Å pore size, 5 µm particle size, Phenomenex) using mobile phase A (0.1% formic acid in water). The separation was carried out using a self-pack NanoLC column (CoAnn Technologies, 75 µm i.d., 30 cm column) with Waters BEH C18 packing material (130 Å pore size, 1.7 µm particle size, Waters Corporation). Separations were performed with a flow rate of 200 nL/min using a 120 min gradient of 1%–75% mobile phase B (ACN + 0.1% formic acid). To prevent carryover, the column was washed with 95%–50% mobile phase B for 20 min and equilibrated with 1% mobile phase B for 30 min before the next sample injection.

The mass spectrometer source was set at 2.2 kV, and the ion transfer capillary was heated to 300°C. The data-dependent acquisition mode was employed to automatically trigger the precursor scan and the MS/MS scans. The MS1 spectra were collected at a scan range of 300–1,800 *m*/*z*, a resolution of 70,000, an automatic gain control (AGC) target of 3 × 10^6^, and a maximum ion injection time of 20 ms. For MS2, the top 12 most intense precursors were isolated with a window of 1.5 *m*/*z* and fragmented by higher-energy collisional dissociation with a normalized collision energy at 30%. The Orbitrap was used to collect MS/MS spectra at a resolution of 17,500, a maximum AGC target of 1 × 10^5^, and maximum ion injection time of 50 ms. Each parent ion was fragmented once before being dynamically excluded for 30 s.

### Analysis of mass spectrometry data

MS/MS automated selected ion chromatogram (MASIC) generator was used to generate selected ion chromatograms (SICs) for all of the parent ions chosen for fragmentation in the LC-MS/MS data (28). MSGF+ software (ver 2024.03.26) (29) was then used to perform peptide searches against the *Mtb* protein database containing 3,994 entries (UniProt for *Mycobacterium tuberculosis* H37Rv, downloaded on 7 March 2021, including the sequence for carbapenem resistance factor [CrfA] Rv2421/Rv2422, downloaded on 12 October 2017) and 16 common contaminant sequences (porcine and bovine trypsin, chymotrypsinogen, human and bovine albumin, and some keratins). Searches were performed with the following parameters: partially and fully tryptic peptides; parent ion tolerance of 20 ppm; methionine oxidation (+15.9949 Da) as a dynamic modification. A spectral probability value of 1.56 × 10^−8^, which was calculated for a 1% false discovery rate, was used for MSGF+ analysis. This value was determined by calculating reverse hits using the forward + reverse (decoy) database (30) for *Mtb*.

Global proteomics analyses were performed using the Multi-Omics Analysis Portal and the pmartR package (31). Potential sample outliers were assessed by a robust Mahalanobis distance (rMd) using the rMd squared values associated with the peptide abundances vector (rMd-PAV), which was applied with all default metrics for proteomics data (correlation, kurtosis, MAD, skewness, and proportion missing) (32). No sample was identified as an outlier, and all samples were included in the analyses. Peptide data were log_2_ transformed and median normalized. Redundant peptides (those mapping to more than one protein) and peptides found in less than two samples were removed before protein rollup. Peptide ion intensities were rolled up to the protein level using the Rrollup method (33) with median centering. Proteins mapped by less than two peptides were removed. Statistical differences in mean protein intensity (label-free quantitation) between groups were assessed via analysis of variance (ANOVA) and independence of missingness (IMD; *G*-test) (34). In cases when a protein was observed in at least two samples in each group, it was considered significantly different if the mean log_2_ intensity between groups had a *P* ≤ 0.05 by ANOVA. In cases when a protein was observed in less than two samples in one group, the number of observed values between groups was considered statistically different if *P* ≤ 0.05 by IMD.

Functional classification of proteins was done using Mycobrowser *Mtb* H37Rv annotation (release 5; 11 July 2024) (35). Data were downloaded from the Mycobrowser website in August 2024 (https://mycobrowser.epfl.ch/releases).

## RESULTS AND DISCUSSION

### Global proteomics analysis

No single *in vitro* model captures all characteristics of dormant *Mtb* found in patients with TB. In our prior work, we have used the Wayne model, a multi-stress model, and carbon starvation (26, 36, 37). For the current work, we used the Grant and Hung model of carbon starvation (5), which induces an antibiotic-tolerant, nonreplicating state through 5-week incubation in a carbon starvation (CS) medium. We cultured six biological replicates of *Mtb* mc^2^6020, a double auxotrophic strain derived from *Mtb* H37Rv (25) to the mid-log phase (replicating, Rep) or under CS. After 5 weeks, CS cultures had entered stasis, defined as minimal growth assessed by optical density (Table S1). We recognize that the proteome of *Mtb* mc^2^6020 could differ from wild-type *Mtb* H37Rv (38). We supplemented both CS and Rep media with lysine and pantothenate, which could have unforeseen effects on the starvation phenotype. Nevertheless, this strain closely models wild-type *Mtb* H37Rv in growth and immunogenicity while being safer for researchers (25).

**Fig 1:**
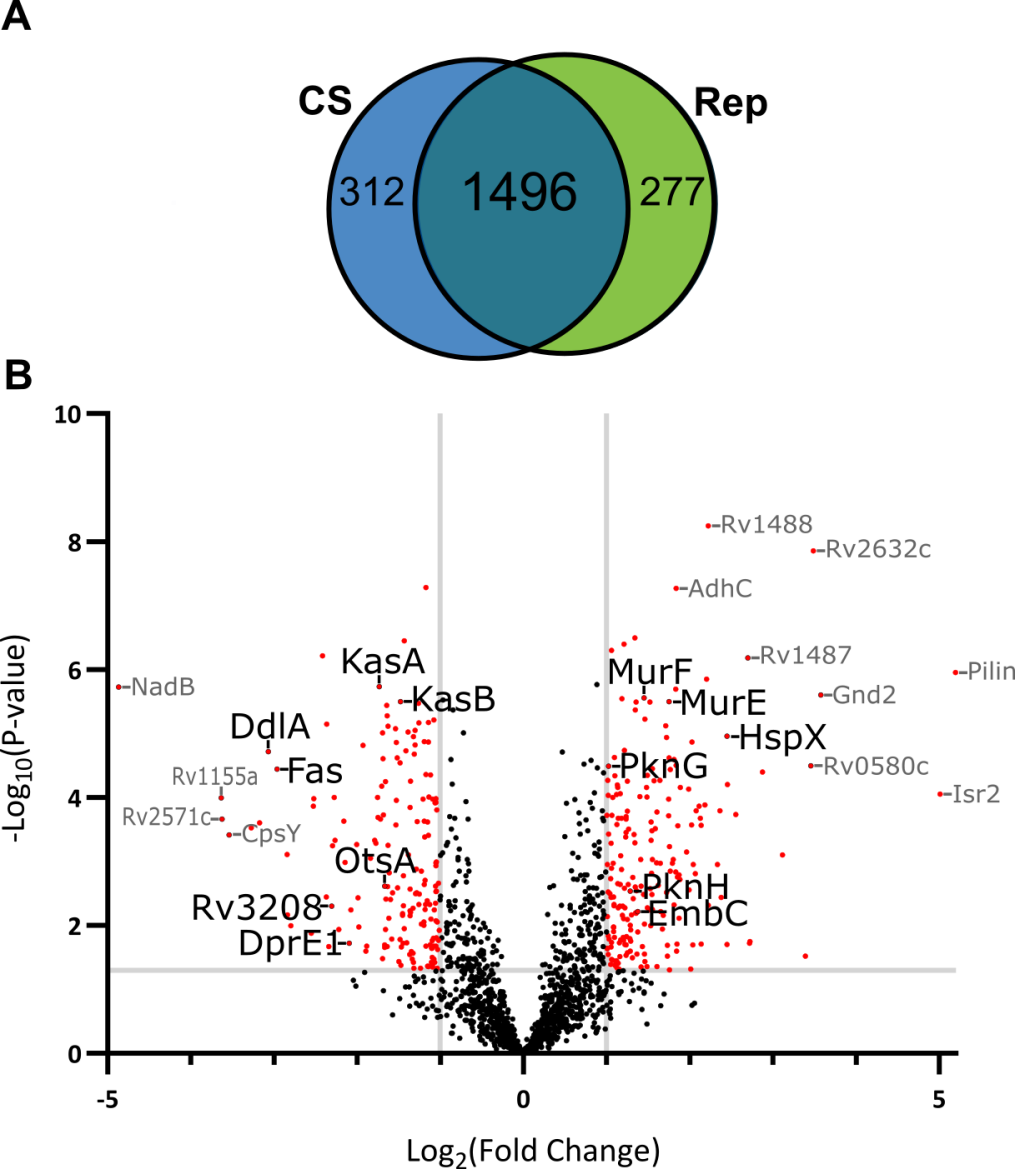
Comparison of proteins identified in CS versus Rep conditions. (**A**) Venn diagram of proteins identified in *n* ≥ 3 samples in CS or Rep conditions. (**B**) Volcano plot of proteins found in CS and Rep conditions. Red dots highlight proteins significantly different in abundance between groups with *P* ≤ 0.05 (ANOVA) and a log_2_ fold change (FC) in CS relative to Rep ≥ 1.0 (more abundant) or ≤−1.0 (less abundant). Proteins identified in only one group (*n* ≥ 3 in group A and *n* ≤ 1 in group B) were not included in the volcano plot due to the inability to calculate an FC. A subset of proteins discussed here is labeled (black font).

Global proteomic profiles of whole-cell lysates were assessed via LC-MS/MS. We identified 2,269 distinct proteins out of the 3,994 annotated proteins in *Mtb* (57% proteome coverage; Table S2). A limitation of bottom-up proteomics is under detection of proteins, particularly of those with low abundance. In *Mtb*, there is over six orders of magnitude difference between the most and least abundant proteins (8, 39). However, the proteome coverage we achieved is on par with other *Mtb* proteomic studies (7, 8, 16, 23). To minimize false positives, we only considered proteins identified in at least three of six replicates of either group (Rep or CS), reducing the overall protein count to 2,085 (Fig. 1A; Table S2). Similar protein counts were identified in Rep (1,773) and CS (1,808) conditions, and most proteins (1,496) were identified in both conditions.

We used spectral intensities to estimate abundance and identify proteins that were differentially abundant between growth conditions. We defined differential proteins as those found to be significantly different (*P* ≤ 0.05) in the presence (*n* ≥ 3 in group A and *n* ≤ 1 in group B, IMD) or abundance (ANOVA, ≥2-fold) across groups. There were 415 proteins enriched in CS, with 43.8% not found in Rep lysates (Fig. 1B; Table S2). Conversely, there were 336 proteins enriched in Rep, with 42.8% not found in CS. There were 1,142 proteins found to be equally abundant in CS and Rep based on these criteria. Overall, 36% of the quantified *Mtb* proteins were significantly altered in response to carbon starvation, indicating robust and broad reprogramming of cellular functions, organization, and processes.

**Fig 2:**
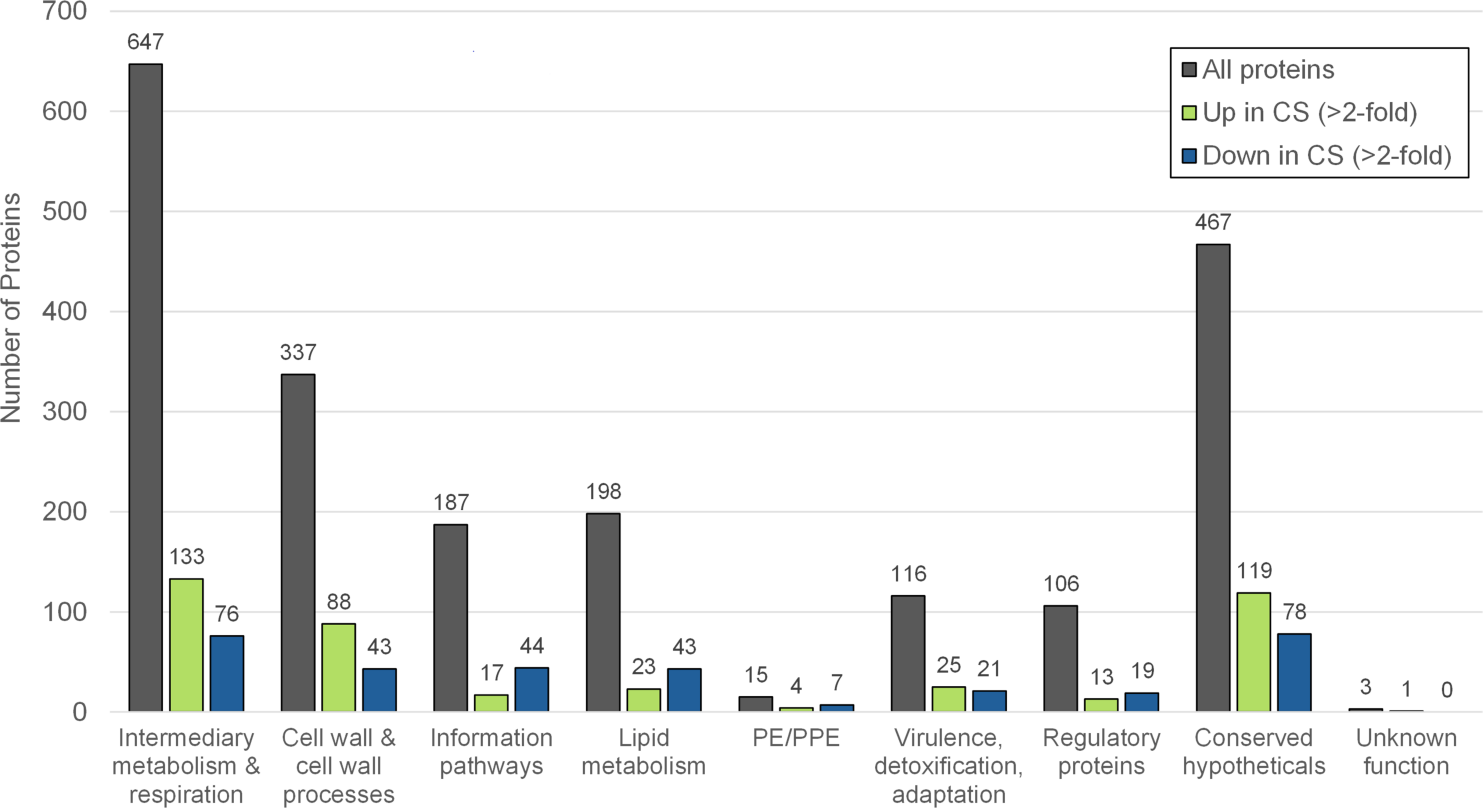
Protein functional classification. Bar chart displaying the distribution of identified proteins across functional categories. The distribution of proteins found in either Rep or CS conditions is shown in gray (all proteins). The distribution of differentially abundant proteins in CS relative to Rep is shown in green (up in CS) and blue (down in CS). Categories were defined and assigned based on Mycobrowser (35) *Mtb* H37Rv (release 5) annotation.

Identified proteins were assigned to functional categories as defined by Mycobrowser (35) (Fig. 2; Table S2). Differentially abundant proteins were found in all functional categories. Most of the proteins that were more abundant in CS were associated with intermediary metabolism and respiration (133; 31%), cell wall and cell wall processes (88; 21%), or conserved hypothetical functions (119; 28%). Proteins that were less abundant in CS were primarily involved in intermediary metabolism and respiration (76; 23%), cell wall and cell wall processes (43; 13%), and conserved hypotheticals (78; 23%). The representation of functional categories within up- and downregulated proteins was widely similar, with no category found to undergo large changes in a certain direction (Fig. S1). The largest categorical shifts with CS were in lipid metabolism (13% down and 5% up) and information pathways (13% down and 4% up).

### Energy metabolism

**Fig 3:**
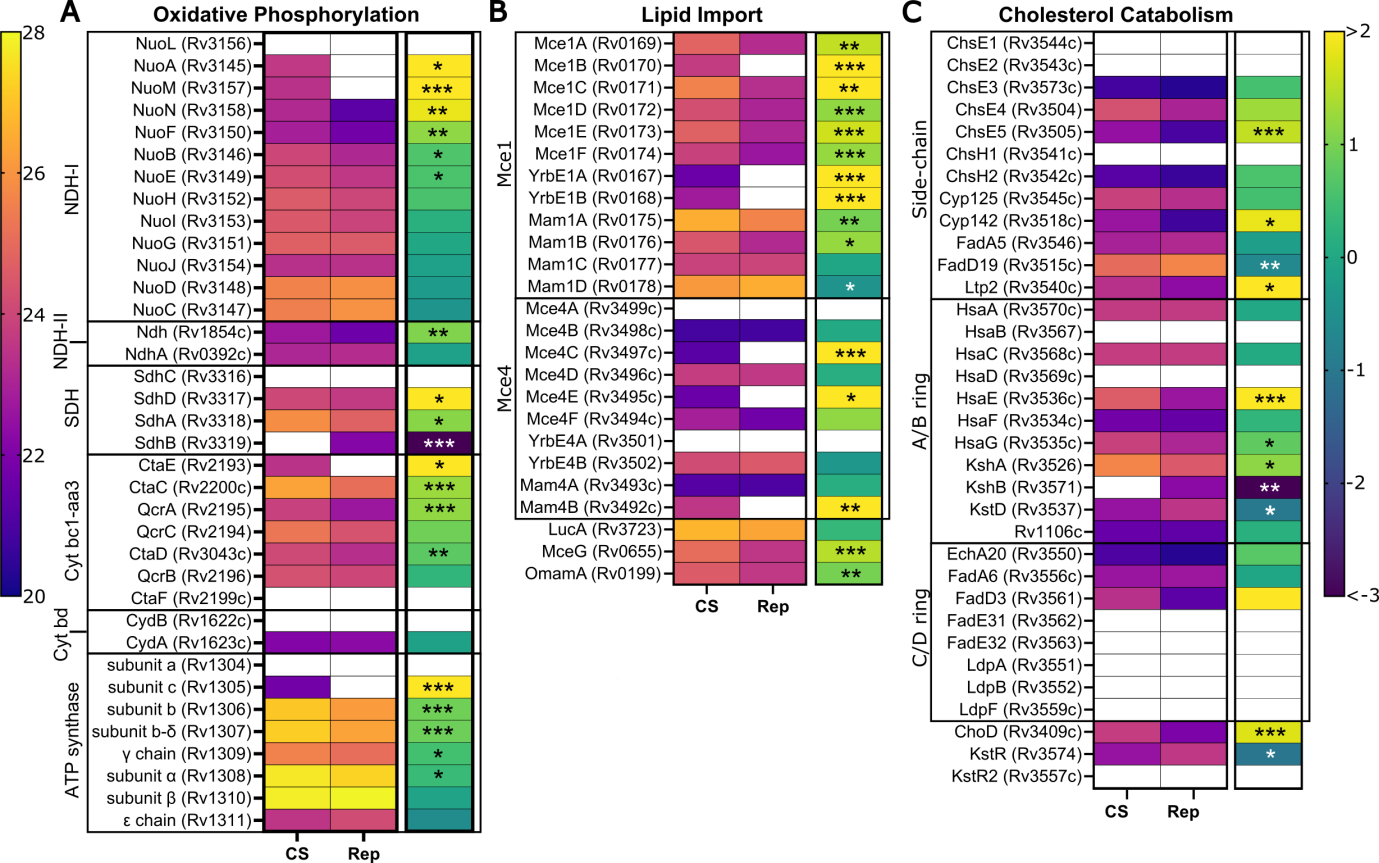
Regulation of energy metabolism in CS versus Rep conditions. Heat maps of mean protein intensities (left map, warm scale) and corresponding log_2_ fold change (right map, cool scale) in CS vs Rep groups. Proteins are identified by UniProt name (locus ID) and are grouped by function: oxidative phosphorylation (**A**), lipid import (**B**), and cholesterol catabolism (**C**). Scale bars are applicable across all corresponding maps. The fold change of proteins identified in only one group (group A: *n* ≥ 3, group B: *n* ≤ 1) was set to the respective maximum value. Asterisks denote significance of the difference in mean intensity between CS and Rep (*: *P* ≤ 0.05, **: *P* ≤ 0.01, and ***: *P* ≤ 0.001). A white box indicates absence of value.

Similar to other studies (5, 13, 23), we observed a dramatic reduction in *Mtb* cellular replication with CS, with little change in optical density (OD_600_) after 5 weeks of culture (Table S1). Others have reported significant reductions in cellular respiration rates and intracellular ATP levels with nutrient-starvation models (13, 22). However, as an obligate aerobe, *Mtb* requires continuous respiration to survive, even in hypoxic and nutrient-starved environments (22, 40). In accordance with these findings, we observed overall retention of oxidative phosphorylation enzymes, with many components of the electron transport chain equally or more abundant in CS (Fig. 3A).

In contrast to findings in hypoxia-induced models of dormancy (41), oxidative phosphorylation appears to progress through the aerobic chain in CS with many components of the cytochrome *bc1-aa3* complex being upregulated. No significant alteration to the terminal oxygen acceptor of the anaerobic chain, cytochrome *bd* oxidase (cyt *bd*), was observed. While increased reliance on cyt *bd* has been associated with *Mtb* adaptation to hostile environments, this reliance appears to be related to the oxygen-deficient and oxidative stress conditions within the host (41, 42). We did not observe this same pattern in our CS model, highlighting a key difference between hypoxia and nutrient-starvation models.

Key components of NADH dehydrogenase type I (NDH-I) and ATP synthase complexes were more abundant in CS. A surprise in our study is that subunit C (Rv1305) the direct target of bedaquiline (43, 44), was only detected in our CS samples. There are eight subunits of the *Mtb* ATP synthase, and subunit C was the only one absent in Rep samples. We hypothesize that subunit C was present below the limit of detection in Rep samples since ATP production via oxidative phosphorylation is required during normal replication.

### Lipid uptake and catabolism

During infections, the primary carbon and energy source for *Mtb* is the co-utilization of host cholesterol and simple carbon fatty acids (45). An interesting finding in our CS model is the apparent upregulation of the Mce1 and Mce4 complexes, which, respectively, import fatty acids and cholesterol into the *Mtb* cell (Fig. 3B). Both of these ABC transporter complexes are composed of six Mce proteins (e.g., Mce1A-F) and two YrbE proteins (e.g., YrbE1A/B) and are associated with different numbers of Mam accessory proteins (46, 47). Every protein of the core Mce1 transporter structure was more abundant in CS. Several of the Mam1 proteins were also more abundant in CS, as well as the ATPase common to all Mce transporters, MceG (Rv0655). The cholesterol transporter Mce4 also appeared more abundant in CS, with Mce4C (Rv3497c) and Mce4E (Rv3495c) only found in CS and Mce4D (Rv3496c) and Mce4F (Rv3494c) found more consistently across the CS group than Rep (Table S2). While the implications of Mce1 complex upregulation are not fully understood, it is known that cholesterol uptake by the Mce4 complex is crucial for the survival of the pathogen in nutrient-starved host environments (48, 49). Our data suggest that *Mtb* compensates for CS by enhancing lipid uptake.

*Mtb*’s dependency on cholesterol in dormancy suggested to us that cholesterol catabolism would be upregulated in CS. *Mtb* uses over 30 enzymes to catabolize cholesterol, as reviewed in references 50, 51. Twelve enzymes degrade the side chain (Cyp125/142, ChsE1/2/3/4/5, ChsH1/H2, FadA5, FadD19, and Lpt2). Of the nine found in our study, three were more abundant in CS (Fig. 3C). Twelve enzymes degrade the A and B rings of cholesterol: ChOX (Rv1106c), KtsD (Rv3537), KshA/B (Rv3526/Rv3571), and HsaA/B/C/D/E/F/G/H. We identified nine of these; two (KstD and KshB) were less abundant, and three (KshA, HsaE, and HsaG) were more abundant in CS. C/D ring catabolism involves at least eight enzymes; most were not found in our study. Catabolism of the A/B ring is negatively regulated by a transcriptional repressor, KstR1 (Rv3574) (52), which was > 2-fold less abundant in CS. Within our data, we identified 53 proteins that are putatively regulated by KstR1. Nineteen were more abundant in CS, including Mce4C, HsaE/G, KshA, and Ltp2. Two KstR1-regulated proteins, OtsB1 (Rv2006) and EchA19 (Rv3516), were identified only in CS, and we suggest that the detection of either of these might confirm an *in vitro* carbon-starvation phenotype (see below). Overall, our data support the important role that cholesterol degradation plays in *Mtb* survival in nutrient-limited environments.

The *Mtb* genome encodes over 300 proteins involved in lipid metabolism (35, 53). There are many lipolytic enzymes, including esterases, lipases, cutinases, and phospholipases (54). Prior work in our group (26) and by others (55, 56) demonstrated that lipase activity is regulated in hypoxia. In the current work, we identified 14 lipases (Lip family members), 1 cutinase (Culp6), and 13 additional esterases (Table S3). Many of these enzymes were differentially abundant in CS (7 up and 13 down), further associating changes in lipid metabolism with stasis.

### Regulation of serine/threonine protein kinases

*Mtb* has eleven serine/threonine protein kinases (STPKs). Recent work by Frando and coworkers demonstrated the importance of the *Mtb* STPKs in widespread *o*-phosphorylation of the proteome (57). Specifically, they showed that over 70% of mycobacterial proteins are *o*-phosphorylated and that 30% of *Mtb* gene expression was changed by perturbing STPK levels. We did not examine phosphorylation of proteins here. However, we detected 9 of the 11 STPKs in our analysis (Table S3). We found that the two essential STPKs, PknA (Rv0015c) and PknB (Rv0014c), were identified in all samples. PknE (Rv1743) and PknJ (Rv2088) were found only in Rep samples, suggesting strong downregulation in CS.

Notably, we found that PknG (Rv0410c) and PknH (Rv1266c) were significantly more abundant in CS. Both of these STPKs are linked to metabolic adaptation and long-term survival in dormancy (58). PknG facilitates survival in hypoxia (59) and under oxidative stress (60). PknG responds to nutrient stress (61) through TCA cycle regulation via phosphorylation of glycogen accumulation regulator A (GarA; Rv1827) (62). GarA was 2-fold more abundant in CS. Furthermore, PknG activity is linked to trafficking and survival in host macrophages (63). PknH was 2.4-fold more abundant in CS. This STPK restricts *Mtb* growth *in vivo* (64). PknH phosphorylates DosR (Rv3133c) and upregulates the DosR regulon in response to nitric oxide (65). Overall, the observed increase in abundance of PknG and PknH aligns with their previously reported roles in mediating the survival of dormant *Mtb*.

### Cell wall biosynthesis is largely retained in CS

*Mtb* has an unusual, lipid-rich cell envelope that provides challenges and opportunities for the treatment of TB (66–68). The mycobacterial envelope is a complex structure with an inner plasma membrane, a cell wall with mycomembrane (MM), and an outer capsule. The cell wall is composed of peptidoglycan (PG), arabinogalactan (AG), and mycolic acids (MA). The outer portion of the cell wall made of MA and trehalose is known as the MM. The layers of the cell wall and envelope are interconnected by a variety of glycolipids and lipoglycans, including phosphatidylinositol mannosides (PIMs), lipomannans (LMs), and lipoarabinomannans (LAMs). Dormant *Mtb* has an altered cell envelope, with loss of acid-fast staining (69). *In vitro* models suggest that the cell wall has reduced MA (9), trehalose monomycolate, and trehalose dimycolate (70, 71), while LMs and LAMs are increased in dormancy (19, 71). *Mtb* PG has both classical 4→3 crosslinks and non-classical 3→3 crosslinks. It is not yet clear if *Mtb* PG crosslinking is altered in carbon starvation, although the PG in replicating cells likely has fewer 3→3 crosslinks than the PG in stationary phase cells (80%) (17) or hypoxic cells (~70%) (72). For these reasons, we assessed the regulation of the various enzymes involved in the biosynthesis of the cell wall in response to CS.

**Fig 4:**
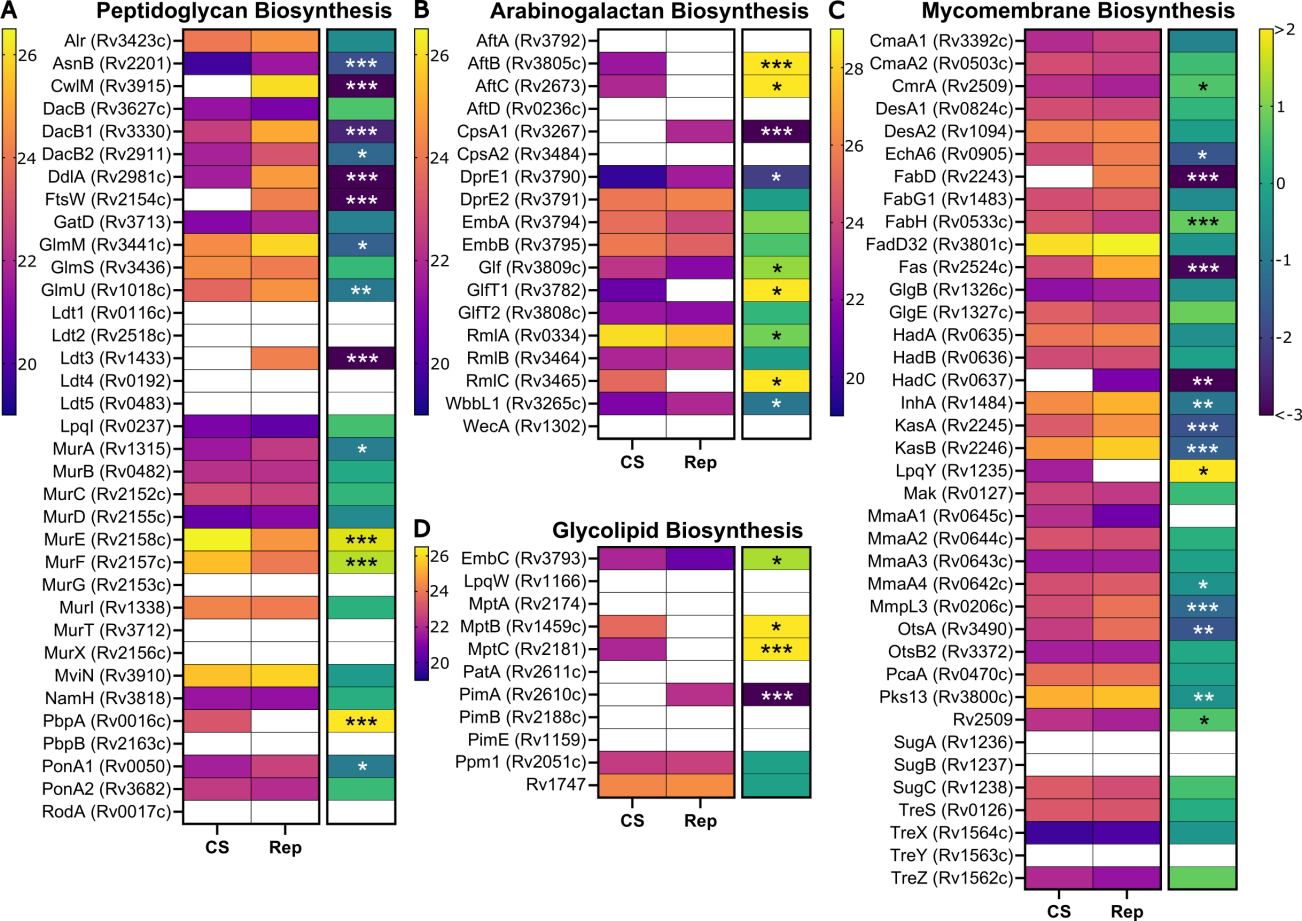
Changes in cell wall biosynthesis in response to CS. Heat maps of mean protein intensities (left map, warm scale) and corresponding log_2_ fold change (right map, cool scale) in CS vs Rep groups. Proteins are identified by Uniprot name (locus ID) and grouped by function: PG biosynthesis (**A**), AG biosynthesis (**B**), MM biosynthesis (**C**), and glycolipid biosynthesis (**D**). Fold change scale bar is applicable across all corresponding maps. The fold change of proteins identified in only one group (group A: *n* ≥ 3 and group B: *n* ≤ 1) was set to the respective maximum value. Asterisks denote significance of the difference in mean intensity between CS and Rep (*: *P* ≤ 0.05, **: *P* ≤ 0.01, and ***: *P* ≤ 0.001). A white box indicates absence of value.

We examined the levels of enzymes involved in PG biosynthesis and found that many were unchanged between CS and Rep except for a few key players (Fig. 4A). PG is composed of a repeating glycan backbone of N-acetylglucosamine (GlcNAc) and N-acetylmuramic acid (MurNAc) interspersed with crosslinked peptide stems. PG biosynthesis starts in the *Mtb* cytoplasm with the synthesis of UDP-GlcNAc conjugated to the peptide stem (D-Ala—D-iso-Gln—*meso*-DAP—D-Ala—D-Ala). The glycan portion is synthesized by GlmS (Rv3436), GlmM (Rv3441c), GlmU (Rv1018c), MurA (Rv1315c), and MurB (Rv0482c); all were identified in both CS and Rep samples. The peptide stem is formed by MurC/D/E/F. Both MurE (Rv2158c) and MurF (Rv2157c) were > 2-fold more abundant in CS. The D-Ala—D-Ala ligase, DdlA (Rv2981c), was less abundant (>8-fold) in CS. DdlA is the target of the anti-TB drug cycloserine (73). Modifications to the nascent PG are mediated by membrane proteins, which are challenging to identify by proteomics. Many of these were not identified in our samples (e.g., NamH, MurX/MraY, MurJ, and MurG). AsnB (Rv2201) was less abundant, and the putative flippase FtsW (Rv2154c) was absent in CS samples. CwlM (Rv3915) regulates MurA activity (74); it was only found in Rep samples.

The remainder of PG biosynthesis occurs in the periplasm, where the activity of various penicillin-binding proteins (e.g., PonA1, PonA2, and PBPA/B), L,D-transpeptidases (e.g., Ldt1-5), and carboxypeptidases (e.g., DacB1/2) produces the final crosslinked PG structure (75). The abundance of most identified penicillin-binding proteins was unchanged. Interestingly, DacB1 and DacB2 were both significantly less abundant in CS. Their function is to remove a D-Ala from the peptide stem, a reaction that precedes Ldt (3→3) crosslinking (72). We speculate that loss of carboxypeptidase activity in CS would result in fewer tetrapeptide stems available for Ldt crosslinking. Only one Ldt, Ldt3 (Rv1433), was detected in our study, and only within Rep samples. Although Betts found that Ldt1 (Rv0116c) was transcriptionally upregulated (17-fold) under nutrient starvation (13), our work and several other global proteomics studies have been unable to detect Ldt1 at the protein level (23, 27). Prior studies characterizing PG crosslinking in replicating and stationary-phase *Mtb* found that 70%–80% of the PG is 3→3 crosslinked in an Ldt-dependent manner (17, 72, 76). To our knowledge, the composition of PG crosslinks in nutrient-starved *Mtb* has not been studied, and our data suggest that further investigation is warranted.

AG is a polymer of branched arabinose chains attached to a “trunk” of galactose (67). AG is covalently attached to both PG and MA. Biosynthesis of AG starts in the cytosol with several enzymes forming the galactan chain: WecA (Rv1302), WbbL (Rv3265c), and GlfT1/T2 (Rv3782/Rv3808c). We found that WbbL was less abundant in CS, while GlfT1 was only observed in CS, suggesting strong upregulation (Fig. 4B). Arabinose is added to the galactose trunks in the periplasm by arabinosyltransferases: AftA (Rv3792), AftB (Rv3805c), AftC (Rv2673), AftD (Rv0236c), and EmbA/B (Rv3794/Rv3795). AftA and AftD were not found in our samples, while AftB and AftC were only found in CS. EmbA, a target of ethambutol, was more abundant (> 2-fold) in CS. Two other relevant AG biosynthetic enzymes are DprE1/E2 (Rv3790/Rv3791). DprE1 was downregulated in CS (> 4-fold) while DprE2, a putative target of pretomanid (77), was unchanged. These findings suggest that AG biosynthesis is altered under CS conditions.

The mycomembrane is an outer membrane layer comprised of MAs (e.g., trehalose mono- and di-mycolate) that form a protective hydrophobic barrier. Many of the enzymes associated with *de novo* MA biosynthesis were less abundant in CS (Fig. 4C). The key fatty acid synthesis enzyme FAS-I (Rv2524c) was strongly less abundant in CS (>7-fold), as was the *fas* transcriptional regulator Rv3208 (approximately 5-fold) (78). Newly synthesized fatty acids are extended by the FAS-II complex of enzymes. Several FAS-II enzymes were less abundant in CS, including KasA/B (approximately 3-fold; Rv2245/Rv2246) and InhA (2-fold; Rv1484). InhA is a target of isoniazid and ethionamide (79). Prior work demonstrated that loss of KasB, with subsequent changes in MA, prevents acid-fast staining, a phenotype observed in latent TB (69, 80). HadC (Rv0637) was only identified in Rep samples; HadA/B (Rv0635/Rv0636) were unchanged. Attachment of MA to AG is mediated by the antigen 85 complex (Rv3804, Rv1886c, and Rv0129c), which was not detected in any of our samples. Trehalose monomycolate, a MA, is transported to the periplasm via MmpL3 (Rv0206c); this essential protein was less abundant in CS (>2-fold). Proteins involved in mycolic acid synthesis have also been found to be downregulated at the transcript and protein level under hypoxic conditions (8, 81). Overall, these patterns across multiple *in vitro* models suggest that it is beneficial to dormant *Mtb* to suppress energy-demanding MA biosynthesis (48, 82).

We observed several CS-related changes in enzymes that synthesize the glycolipids and lipoglycans of the inner and outer membrane: PIMs, LMs, and LAMs (Fig. 4D). It has been proposed that PIMs are less abundant in stasis, while LMs and LAMs are more abundant (19). We saw results consistent with this hypothesis in our data. The α-mannopyranosyl transferase that initiates PIM biosynthesis, PimA (Rv2610c), was only found in Rep samples, suggesting strong downregulation with CS. The mannosyltransferases MptB (Rv1459c) and MptC (Rv2181), enzymes that are involved in the processing of PIMs to LMs, were both only found in CS samples. Additionally, the arabinotransferase EmbC (Rv3793), which modifies mature LM with arabinose to form LAMs (67, 83), was significantly more abundant in CS (5.5-fold).

### Targets of anti-TB drugs

Drug treatment for TB is long, usually lasting at least 6 months (1, 4). Monotherapy is not used for TB due to low efficacy and the high probability of selecting drug-resistant mutants. The most common treatment regimen for drug-susceptible TB includes isoniazid, rifampicin, pyrazinamide, and ethambutol. For years, the treatment of drug-resistant TB varied from patient to patient, with generally poor outcomes (15%–40% patient mortality globally) (1). Treatment outcomes for drug-resistant TB improved in 2022, with approval of the combination therapy of bedaquiline, pretomanid, and linezolid (termed BPaL) (84, 85). Other drugs used to treat TB include fluoroquinolines, rifapentine, cycloserine, streptomycin, kanamycin, and (rarely) β-lactams (73). Currently, the standard treatment for LTBI is rifampicin, rifampicin plus isoniazid, or rifapentine plus isoniazid (86).

Nearly all (99.9%) of *Mtb* are killed within the first 2 weeks of treatment (87). The remaining months of treatment are implemented to kill non-replicating *Mtb* that is particularly challenging to eradicate. Many studies have found that antibiotics have decreased potency against dormant *Mtb* (5, 13, 21, 88, 89). This phenomenon is termed phenotypic drug resistance and is a hallmark of *in vitro* dormancy models (12). A 2017 review summarized findings from the literature and found that rifampicin, rifapentine, metronidazole, bedaquiline, pretomanid, and fluoroquinolines are the most active drugs against non-replicating *Mtb* (90). Studies have found that nutrient-starved *Mtb* is more drug tolerant than hypoxic *Mtb* (5, 13, 22, 89). For example, rifampicin is ~50-fold less potent against nutrient-starved *Mtb* compared to hypoxic *Mtb* (22).

**Fig 5:**
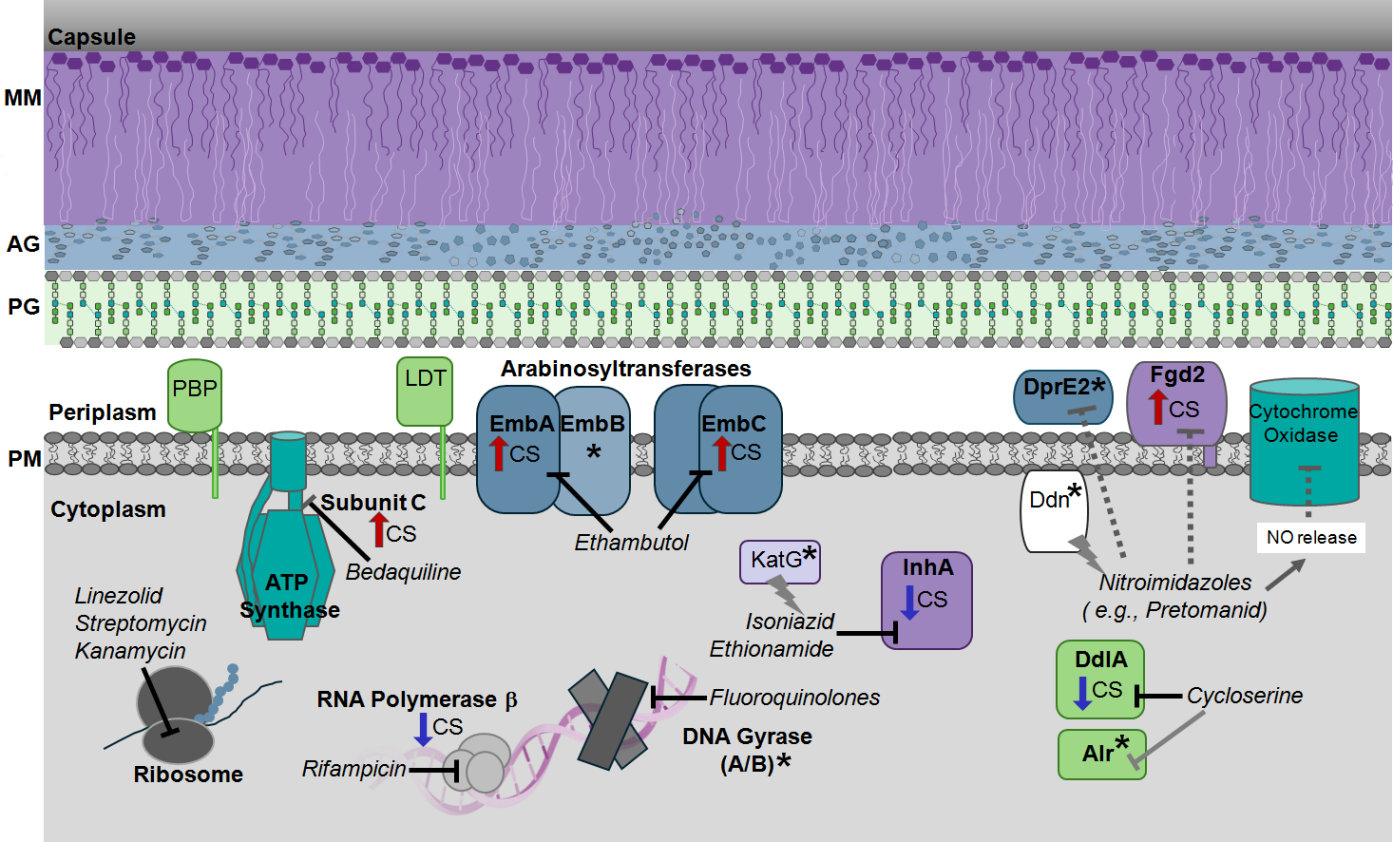
The targets of clinically approved TB drugs are present in *Mtb* under CS and Rep conditions. Drug targets involved in cell wall biosynthesis are color-coded by functional localization: MA/MM (purple), AG (blue), and PG (light green). Proteins involved in transcription and translation are shown in gray. Other relevant drug targets are shown in teal. Altered protein levels in CS are indicated by arrows: red (more abundant) and dark blue (less abundant). An asterisk indicates no significant change in protein levels between CS and Rep. Drug names are italicized. Nitroimidazoles have a complex mechanism and target more than one pathway, as indicated.

We analyzed our proteomic data for the relative levels of drug targets in CS and Rep *Mtb* samples (Fig. 5). The *Mtb* cell wall is a target of many antibiotics. Synthesis of mycolic acids is inhibited by isoniazid and ethionamide, which both target an enoyl-[acyl-carrier-protein] reductase (InhA; Rv1484) (79). This enzyme was found in both CS and Rep samples, as noted above, but was less abundant in CS (2-fold). The levels of the enzymes that activate isoniazid and ethionamide, KatG (Rv1908c) and EthA (Rv3854c), respectively, were unchanged. A trehalose monomycolate exporter, MmpL3 (Rv0206c), is the target of SQ109 (91), a drug that is still under evaluation for the treatment of TB. It was >2-fold less abundant in CS.

Nitroimidazoles (e.g., pretomanid and delamanid) have a complex mechanism of action (92). Pretomanid is effective in replicating and non-replicating *Mtb*. In hypoxia, pretomanid is activated by deazaflavin-dependent nitroreductase (Ddn; Rv3547) and produces reactive nitrogen species (e.g., nitric oxide, NO), which inhibit cytochrome oxidase (92). Pretomanid may also inhibit an F420-dependent hydroxymycolic acid dehydrogenase (Fgd2; Rv0132c) (93). This enzyme is involved in MA biosynthesis and was only identified in our CS samples. Pretomanid may inhibit DprE2 (Rv3791) (77), an essential enzyme that forms a precursor for AG. Protein levels of Ddn and DprE2 were unchanged in CS versus Rep conditions.

The arabinosyltransferases EmbA (Rv3794), EmbB (Rv3795), and EmbC (Rv3793) polymerize arabinose during AG biosynthesis and are inhibited by ethambutol, a front-line drug. Prior studies found that ethambutol was less effective in nutrient-starved and hypoxic *Mtb* (20, 88, 89). The A and C subunits were both >2-fold more abundant in CS. Further studies are needed to determine if upregulation of these enzymes contributes to ethambutol resistance. Cycloserine targets PG biosynthesis through inhibition of the D-alanine—D-alanine ligase (DdlA; Rv2981c) and, to a lesser extent, alanine racemase (Alr; Rv3423c) (73). DdlA was highly less abundant in CS (> 8-fold). This is consistent with the finding by Xie et al. that cycloserine was not effective in killing nutrient-starved *Mtb* (89). PG biosynthesis is disrupted by β-lactams, although this therapeutic class is only rarely used to treat TB (94, 95). Targets of carbapenems, a sub-class of β-lactams, include penicillin-binding proteins and Ldts. The abundance of these enzymes in CS is summarized in Fig. 4A.

Some drugs target the pathogen’s transcription and translation (73). Fluoroquinolones (e.g., moxifloxacin) target DNA gyrase A and B (Rv0005 and Rv0006) (96); both were not significantly changed between CS and Rep samples. Rifampin inhibits the RNA polymerase β-subunit (Rv0667). This protein was less abundant in CS (2-fold); prior work found rifampin resistance in nutrient-starved conditions (13). Streptomycin, kanamycin, and linezolid all target ribosomal RNA complexes (73), which were not quantified in our study.

Bedaquiline is a diarylquinoline that targets ATP synthase, disrupting energy metabolism (43, 44, 97). More specifically, it binds to subunit-c (Rv1305) of the ATP synthase F_0_ domain. We found that subunit-c was more abundant in CS. Prior studies found that dormant bacilli have increased susceptibility to bedaquiline (97). It would be worth investigating the relationship between regulation, abundance, and activity of ATP synthase and bedaquiline efficacy.

Lastly, pyrazinamide is a pro-drug activated by the pyrazinamidase PncA (Rv2043c) (98). We did not detect this enzyme in our samples. Additionally, there are reports that this drug acts on pantothenate biosynthesis (99). We used an auxotrophic strain with disruption in two genes that make pantothenate (PanC and PanD) (25). Therefore, we could not assess the regulation of these targets in our study.

Before new drugs and drug regimens are approved, it is critical to assess how effective treatments are against dormant *Mtb*. To summarize, we found that the targets of many drugs used to treat TB are present in both Rep and CS samples. We describe changes in protein abundance that could have implications for drug susceptibility of dormant *Mtb*. We view this information as supplemental to direct measurements of drug potency in cells and animal models, since many factors influence efficacy besides target abundance, including efflux pumps and cell wall permeability (88).

### Comparison with prior starvation -omics studies

To our knowledge, no previous study has thoroughly characterized whole-cell proteomic changes in *Mtb* in response to extended CS. Our findings presented here fill this gap and provide insight relevant to dormant *Mtb*. Two prior studies conducted proteomics analysis of *Mtb* under CS (13, 23), albeit with major limitations. Betts and coworkers investigated proteomic changes in *Mtb* in response to short-duration nutrient starvation (5 days); however, they identified only seven proteins as differentially regulated (13). Matching their findings, we found Tig (Rv2462c) and GrpE (Rv0351) to be downregulated and Rv2557 and HspX (Rv2031c) to be upregulated in CS. We saw conflicting results for two other proteins (Rv1860 and Rv1980c). The remainder of their ground-breaking work focused on transcriptional changes after 4–96 hours of CS; we will not discuss those results here because of the differences in study design. There have been many advances in MS-based proteomics since the early 2000s that have greatly improved the power of peptide detection. Still, more recent investigations into nutrient-starved *Mtb* have limitations. The Grant et al. CS study (5) did not include proteomic analysis, and the most recent CS study that did was published by Albrethsen et al. in 2013 (23). Albrethsen induced dormancy using the Betts nutrient deprivation model (i.e., standing cultures were grown for 6 weeks in PBS). They isolated proteins from culture filtrates for identification using LC-MS/MS analysis but did not analyze whole cell lysates, a limitation of their study.

**Fig 6:**
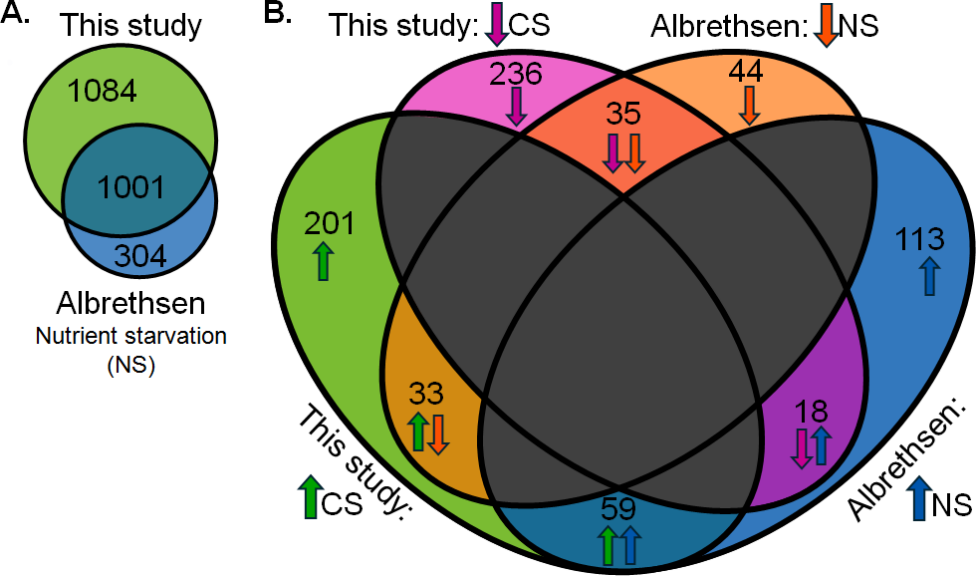
Comparative analysis of proteins associated with carbon or nutrient starvation. (**A**) Total shared and unique *Mtb* proteins were identified in at least three replicates (our study) or two replicates (23, 23) of either starved or Rep conditions. (**B**) Comparison of differentially expressed proteins between the two studies. There were 59 proteins that were upregulated (FC > 2), and 35 proteins were downregulated (FC > 2) in CS in both studies; 51 proteins were discordant between the studies.

Despite significant differences in study design, we compared our CS proteomic profile to that from the Albrethsen study (23) (Fig. 6). As expected for analyzing cellular proteins compared to secreted proteins, we achieved higher proteome coverage (52% vs 33%, respectively). There were 1,001 proteins identified in both of our studies (Fig. 6A). In Albrethsen’s work, of the 1,305 proteins that were identified in at least two samples from either CS or Rep (*N* = 3), there were 343 upregulated (>2-fold; *P* < 0.05) and 288 downregulated (> 2-fold; *P* < 0.05) in CS. By comparison, we identified 415 upregulated (>2-fold; *P* < 0.05) and 336 downregulated (>2-fold; *P* < 0.05) proteins. These results are illustrated in Fig. 6B. There were 59 upregulated and 35 downregulated proteins identified in both studies, which we consider high-confidence CS-responsive proteins (Table S4). There were 51 proteins that showed opposite regulation; we term these discordant. Just over 9% of proteins identified across both studies were concordant. We conclude that the lack of proteomics analyses of the CS model over the last decade highlights the current study as a significant resource.

### Protein indicators of the carbon-starved phenotype

Specific protein indicators of dormancy, especially for CS, are variable and poorly defined (18). For example, a putative biomarker for dormancy is HspX (Rv2031c). Also named alpha crystalline, this protein is a chaperone induced during CS (13) and hypoxia (7, 100). Betts described the upregulation of the select isoforms of HspX under CS (13). However, the use of HspX as a dormancy biomarker should be done with extreme caution. It is highly expressed in Rep cultures and is the second most abundant protein in the *Mtb* proteome (39). HspX was >5-fold more abundant in our CS samples but was also present in all Rep samples. Lastly, Albrethsen et al. did not detect a significant change in HspX in their CS study (23). Unless a dormancy-associated isoform of HspX is characterized and validated, we would not recommend HspX as an indicator of dormancy.

We sought to create a list of proteins that are specifically associated with an *in vitro* nutrient-starved phenotype. From our data, we considered the 182 proteins found only in CS and absent in Rep. We compared this list to proteins that were at least 10-fold upregulated in Albrethsen’s CS model to identify common proteins (23). From this analysis, we propose that Fgd2 (Rv0132c), Rv0571c, Rv1019, Gmk (Rv1389), CysG (Rv2847c), FadD13 (Rv3089), EchA19 (Rv3516), and Rv3618 are likely specific to the *in vitro* carbon-starved phenotype.

The DosR regulon plays a key role in non-replicating *Mtb* induced by hypoxia (100, 101). Although we identified 24 DosR-regulated proteins in our study, only nine were more abundant (> 2-fold) in CS. Among those, four DosR-regulated proteins—Rv0571c, Rv2004c, OtsB1 (Rv2006), and Rip3 (Rv2625c)—were present in our CS samples and absent in Rep samples. We propose that these are potential indicators of a non-replicating or dormant state, although only one (Rv0571c) was identified as significantly upregulated in Albrethsen’s study (23). None of the components of the regulon’s activation system, DosS (Rv3132c), DosT (Rv2027c), and DosR (Rv3133c), were significantly changed between our CS and Rep samples.

Overall, there are 11 proteins that we propose are specific to the *in vitro* CS phenotype in *Mtb*: Fgd2, Rv0571c, Rv1019, Gmk, CysG, FadD13, EchA19, Rv3618, Rv2004c, OtsB1, and Rip3. Four of the proteins are implicated in intermediary metabolism and respiration: Fgd2, Gmk, CysG, and Rv3618. Among these, Fgd2 is intriguing because it produces keto-mycolic acids (93), a component of the cell wall. It is an F420-dependent hydroxy-mycolic acid dehydrogenase and a putative target of the drug pretomanid (93). The functional significance of upregulation of Fgd2 is unclear, but it could be involved in remodeling mycolic acids in dormancy. For example, Fgd2 might play a role in the increase of a C_77_ keto-mycolic acid in dormancy (9).

FadD13 and EchA19 are both linked to lipid metabolism. FadD13 is a long-chain fatty acyl co-A synthetase implicated in the maintenance of mycolic acids, including under acidic conditions (102, 103). EchA19 is an enoyl-CoA hydratase involved in cholesterol degradation (104). OtsB1 (Rv2006) is a part of the DosR regulon and may participate in trehalose biosynthesis. Although OtsB1 has yet to be biochemically characterized, it is a putative trehalose-6-phosphate phosphatase with homology to the essential enzyme OtsB2 (Rv3372) (105). A 2004 paper claiming that OtsB1 lacked phosphatase activity provided no data to support that conclusion (106). As noted above, OtsB1 and EchA19 are both KstR1-regulated proteins (52).

We note that several proteins we identified as CS specific are linked to the cell wall components: Fgd2, FadD13, and OtsB1. It is tempting to speculate that these enzymes facilitate the remodeling of the cell wall for survival in dormancy. Further studies are warranted. As far as we could determine, there are no antibodies available for these proteins, which would be useful research tools for validating the CS phenotype in *Mtb*.

### Conclusions

There are several *in vitro* models that induce a non-replicating state in *Mtb* that mimics bacterial physiology observed during latent TB infections. In the current study, we used carbon starvation to model *Mtb* dormancy. We report the first in-depth analysis of *Mtb* whole-cell proteomic changes in response to CS. We suggest a set of proteins specific to the *in vitro* CS state that could be used to verify starvation phenotypes in future studies.

Many of our findings align with previous reports of hypoxia-induced changes, strengthening our understanding of the *Mtb* non-replicative state. For example, we identified increased PknG and PknH abundance, kinases previously shown to play a central role in responding to external stressors (e.g., hypoxia [59] and oxidative stress [60, 65]). Additionally, we confirmed that carbon starvation, like hypoxia, influences cell wall biosynthesis with measurable downregulation of mycomembrane-associated biosynthetic machinery. This result is consistent with effects on *Mtb*’s cell wall and cell wall staining observed in patient samples (69). However, we also observed key differences in protein regulation between CS and previous hypoxia-related findings. For example, energy metabolism appears to proceed through the aerobic chain in CS. We also found evidence that lipid uptake and catabolism are upregulated in CS, which has not been described for hypoxia models. Furthermore, the DosR regulon appears much less central to CS-induced reprogramming than in hypoxia.

Considering the difficulty of modeling the complex and multifaceted environment that dormant *Mtb* are exposed to *in vivo*, these differences among models are to be expected. Overall, the ability to compare proteomic regulation in *Mtb* across multiple dormancy models broadens our insight into the strengths of said strategies and challenges us to continue to develop better models for studying this deadly pathogen in the laboratory.

## Data Availability

Proteomics data have been deposited in MassIVE (accession code: MSV000096247).

## SUPPLEMENTAL MATERIAL

Supplemental material msystems.01530-24-s0001.docx Table S1 and Figure S1.

Table S2 msystems.01530-24-s0002.xlsx Identified proteins and relative abundance.

Table S3 msystems.01530-24-s0003.xlsx Protein information for categories and heat maps.

Table S4 msystems.01530-24-s0004.xlsx Comparison between Devlin et al. and Albrethsen et al.

ASM does not own the copyrights to Supplemental Material that may be linked to, or accessed through, an article. The authors have granted ASM a non-exclusive, world-wide license to publish the Supplemental Material files. Please contact the corresponding author directly for reuse.
