# Supplementary material for: Proteomic characterization of Mycobacterium tuberculosis subjected to carbon starvation
Source: mSystems. 2025 Apr 15;10(5):e01530-24. doi: 10.1128/msystems.01530-24 (PMC12090744; doi:10.1128/msystems.01530-24)
Supplement: Supplemental material — Table S1 and Figure S1. [file msystems.01530-24-s0001.docx]

**Supporting Information for:**

**K.L. Devlin et al. “Proteomic Characterization of M. tuberculosis subjected to Carbon Starvation.**

**Table S1**. Culture growth under CS and Rep conditions for *M. tuberculosis* (mc^2^6020).

| **Culture** | **Starting OD_600_** | **Growth Time (d)** | **Harvest OD_600_** | **Lysate Conc (μg/mL)** |
| --- | --- | --- | --- | --- |
| CS- A | ~0.2 | 35 | 0.31 | 803 |
| CS- B | ~0.2 | 35 | 0.31 | 859 |
| CS- C | ~0.2 | 35 | 0.30 | 749 |
| CS- D | ~0.2 | 35 | 0.29 | 571 |
| CS- E | ~0.2 | 35 | 0.29 | 733 |
| CS- F | ~0.2 | 35 | 0.31 | 800 |
| Rep J | ~0.2 | 5 | 1.04 | 4178 |
| Rep K | ~0.2 | 5 | 0.93 | 3957 |
| Rep L | ~0.2 | 5 | 1.00 | 3812 |
| Rep M | ~0.2 | 5 | 0.92 | 2500 |
| Rep N | ~0.2 | 5 | 0.96 | 3423 |
| Rep O | ~0.2 | 5 | 1.10 | 3198 |

**Figure S1**. Functional classification of proteins differentially regulated under CS conditions.
